# Synthesis and Thermoelectric Properties of Bi_2_Se_3_ Nanostructures

**DOI:** 10.1007/s11671-010-9795-7

**Published:** 2010-10-01

**Authors:** K Kadel, Latha Kumari, WZ Li, Jian Yu Huang, Paula P Provencio

**Affiliations:** 1 Department of Physics, Florida International University, Miami, FL 33199, USA; 2 Sandia National Laboratories, Center for Integrated Nanotechnologies (CINT), Albuquerque, NM 87185, USA

**Keywords:** Bismuth selenide, Nanoflakes, Solvothermal synthesis, Power factor, Figure of merit

## Abstract

Bismuth selenide (Bi_2_Se_3_) nanostructures were synthesized via solvothermal method. The crystallinity of the as-synthesized sample has been analyzed by X-ray diffraction, which shows the formation of rhombohedral Bi_2_Se_3_. Electron microscopy examination indicates that the Bi_2_Se_3_ nanoparticles have hexagonal flake-like shape. The effect of the synthesis temperature on the morphology of the Bi_2_Se_3_ nanostructures has also been investigated. It is found that the particle size increases with the synthesis temperature. Thermoelectric properties of the Bi_2_Se_3_ nanostructures were also measured, and the maximum value of dimensionless figure of merit (ZT) of 0.096 was obtained at 523 K.

## Introduction

Thermoelectric (TE) materials are considered as critical components for solid-state power generating and refrigerating devices [1]. However, the state-of-the-art bulk thermoelectric material is only 30% efficient as the refrigerating material when compared to the Freon-based conventional refrigerating material [2]. Hence, their relatively low energy conversion efficiency limits the practical application of TE material as a power generator and/or refrigerator. The most important issue of the thermoelectric research is to increase the efficiency of thermoelectric materials. The efficiency of TE material can be defined by dimensionless thermoelectric figure of merit (ZT),

$$\text{ZT}=\frac{S^{2}\sigma }{k}T$$

where S is Seebeck coefficient, σ is electrical conductivity, Κ is thermal conductivity, and T is absolute temperature at which figure of merit is measured. The quantity S^2^σ is most commonly referred as power factor. Increase in power factor and decrease in thermal conductivity are required for the enhancement of ZT value. Theoretical predictions and experimental results show that a nanostructured low-dimensional TE material can exhibit high thermoelectric efficiency [3-5]. Nanostructures can induce the reduction in thermal conductivity by the interface or boundary scattering of phonons and the increment in power factor by quantum confinement of electrons [3,6]. In the recent years, researchers have shown increased interest to investigate thermoelectric properties of various solid-state TE materials in their nanostructured form. Recently, a high ZT of about 2.5 for Bi_2_Te_3_/Sb_2_Te_3_ superlattices has been reported at 300 K [7].

According to Slack [8], semiconductors having narrow band gap and high mobility carriers are best suited as thermoelectric materials. Bismuth selenide (Bi_2_Se_3_) is a V-VI semiconductor with a narrow band gap of about 0.3 eV [9,10], which has potential application in optical recording system [11], photoelectrochemical devices [12], and thermoelectric devices [9,10]. In recent years, bismuth chalcogenides gained much research interest due to their good thermoelectric properties and high ZT values at room temperature [7,13].

Over the years, a wide variety of synthesis techniques have been developed to synthesize various nanostructures of Bi_2_Se_3_. Wang et al. [14] reported low-temperature solvothermal method to obtain Bi_2_Se_3_ nanostructures in ethylenediamine (EN), Giani et al. [15] used chemical vapor deposition method to synthesize Bi_2_Se_3_ thin film, and Jiang et al. [16] synthesized Bi_2_Se_3_ nanosheets by microwave heating in the presence of ionic liquid. Among the various synthesis techniques employed for the formation of Bi_2_Se_3_ nanostructures, the solvothermal/hydrothermal process is attracting much interest due to the advantages of high yield, low synthesizing temperature, high purity, and high crystallinity. Xie et al. [17] and Yu et al. [18] synthesized Bi_2_Se_3_ nanostructures using ethylenediamine (EN) as solvent, and Batabyal et al. [19] reported synthesis of Bi_2_Se_3_ nanorods using dimethyl formamide (DMF) as solvent. Solvothermal/hydrothermal process has been successfully employed to synthesize different nanostructures of Bi_2_Se_3 _[20,21], and [22]. To the best of authors' knowledge, measurement of thermoelectric properties of Bi_2_Se_3_ is seldom reported. Recently, Lin et al. [23] reported the thermoelectric measurement of nanostructured Bi_2_Se_3_ obtained from decomposition of the single-source precursor. In this communication, we report the synthesis of flake-like Bi_2_Se_3_ nanostructures via solvothermal route in DMF at various synthesis temperatures for different durations. The effect of the synthesis temperatures on the structure and morphology of the Bi_2_Se_3_ nanostructures has been investigated. TE properties of the Bi_2_Se_3_ nanostructures have also been measured and found superior to their bulk counterpart.

## Experimental

Analytically pure bismuth nitrate pentahydrate (Bi(NO_3_)_3._5H_2_O, Fisher Scientific) and selenium (Se, Acros) powder were used as precursor materials for the synthesis of Bi_2_Se_3_, and 1 mmol of Bi(NO_3_)_3._5H_2_O and 1.5 mmol of Se powder (in molar ratio of 2:3) were measured and added into a Teflon-liner. Then, 4 mmol of sodium hydroxide (NaOH, Acros) as a pH-controlling and pH-reducing agent, and 2 mmol of ethylenediaminetetraacetic acid (EDTA, Acros) as a shape-directing additive were added. Later, the Teflon-liner was filled up to 80% of its total volume with DMF and was placed in an ultrasonicator for 30 min to obtain a uniform reaction mixture. After the sonication, the Teflon-liner was placed in an autoclave and sealed tightly. Then, the autoclave was heated in the furnace at 140 and 200°C for 24 h. After the synthesis, the autoclave was allowed to cool down to room temperature naturally. The black precipitate resulted from the reaction was vacuum filtered, rinsed with ethanol and distilled water several times, and dried at 100°C in vacuum for 4 h to get the sample in powder form. Samples prepared in DMF at 140 and 200°C for 24 h are termed as BiSe-1 and BiSe-2, respectively. In order to measure the TE properties of the material, a large amount of as-prepared BiSe-2 powder sample was annealed in the presence of hydrogen and argon for 4 h before TE properties were measured.

X-ray diffraction (XRD) measurements were taken using Siemens D5000 diffractometer equipped with a Cu anode operated at 40 kV and 40 mA. The XRD patterns were collected with a step size of 0.01° and a scan rate of 1 step/s. Surface morphology analysis was performed by a field-emission scanning electron microscope (SEM, JEOL JSM-6330F, 15 kV). Transmission electron microscopy (TEM) images, selected-area electron diffraction (SAED) patterns, and energy dispersive X-ray spectroscopy (EDS) spectrum were obtained from FEI Tecnai F30 apparatus operated at an accelerating voltage of 300 kV with a point-to-point resolution of 2Å.

For TE properties measurement, the powder sample BiSe-2 was pressed at 500°C in graphite dies with a 12.7 mm central cylindrical opening diameter using a dc hot-press method to obtain cylindrical bulk discs. Since the pressure applied to the sample is very high (~80 MPa), these bulk samples are highly dense. The measured density of the sample by using Archimedes's principle is 6.59 gcm^-3^, which is at around 97% of the material's theoretical density (6.798 gcm^-3^). These bulk samples were then cut into 2 mm × 2 mm × 12 mm bars for four-probe electrical conductivity and Seebeck coefficient measurements and also into 12.7-mm-diameter discs with appropriate thickness for the thermal conductivity measurement. The electrical conductivity and Seebeck coefficient were measured by using commercial equipment (Ulvac, ZEM-3) from room temperature to 523 K, and the thermal conductivity was measured by using a laser flash system (Netzsch LFA 457) from room temperature to 523 K.

## Result and Discussion

### Structure Characterization of Bi_2_Se_3_ Nanoparticles

**Figure 1:**
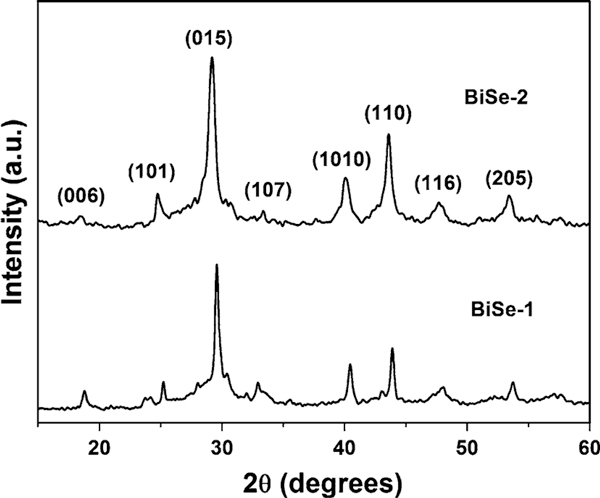
**XRD patterns of the as-prepared Bi_2_Se_3_ samples synthesized in DMF for 24 h, BiSe-1 at 140°C, and BiSe-2 at 200°C**.

Figure 1 shows the XRD patterns of the samples prepared in DMF for 24 h at 140°C (BiSe-1) and 200°C (BiSe-2), respectively. The peaks in the XRD pattern can be indexed as rhombohedral Bi_2_Se_3_ (JCPDS: 033-0214) with space group R$\bar{3}$m(166). The strong (015) diffraction peak represents the prominent growth orientation of Bi_2_Se_3_ nanoparticles along the [015] direction. The sharp peaks in the XRD profiles indicate the high crystallinity of the as-prepared Bi_2_Se_3_ samples. No peaks for other elements were detected, indicating the high purity of the Bi_2_Se_3_ samples.

**Figure 2:**
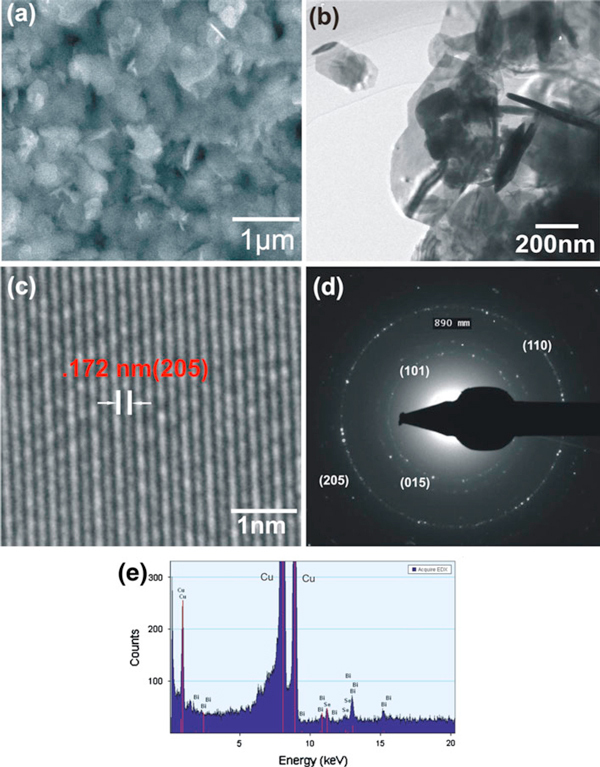
**SEM and TEM images of the as-prepared Bi_2_Se_3_ samples synthesized in DMF at 140°C for 24 h (BiSe-1)**. (**a**) SEM image, (**b**) TEM image, (**c**) HRTEM image, (**d**) SAED pattern, and (**e**) EDS pattern.

The surface morphology and structure of the as-prepared samples were analyzed by SEM and TEM. Figure 2 shows the SEM and TEM examination results of the sample BiSe-1. The SEM image (Figure 2a) shows thin and appreciably fine flake-like nanostructures of Bi_2_Se_3_ with an average size of 300 nm. The process of formation of Bi_2_Se_3_ nanoflakes can be attributed to the layered nature of Bi_2_Se_3_ structure [24,25]. A typical TEM image (Figure 2b) reveals that the as-prepared sample consists of the nanoflakes of sizes ranging from 140 to 380 nm. The isolated nanoflake is very thin and translucent to electron beams when examined by TEM. A previous work by Wang et al. [20] also reported on the solvothermal synthesis of flake-like crystal of Bi_2_Se_3_ in diethyl glycol at 160°C for 22 h. The size of the flake was in the range of 200–400 nm, which is comparable to the size of the nanoflakes reported in the present work. It should be pointed out that although both the solvent and the temperature are different between our work and the reported work (ref. 20), the Bi_2_Se_3_ nanoparticles from the two methods have similar morphology and size, indicating that the solvents (diethyl glycol and DMF) and a temperature in the range of 140–200°C have the same or similar effect on the formation of the Bi_2_Se_3_ nanoflakes. A large repertoire of solvents and a broad range of temperature will provide us some flexibility in the selection of the synthesis conditions of this type of material. Figure 2c shows a high-resolution TEM image of a Bi_2_Se_3_ nanoflake. The lattice fringes are clearly distinguishable, and calculated lattice spacing of 0.172 nm is in agreement with the *d*-spacing of the (205) planes of rhombohedral Bi_2_Se_3_. Figure 2d shows the clearly distinguishable SAED ring patterns that can be indexed to different lattice planes of rhombohedral Bi_2_Se_3_. The chemical composition of the as-prepared Bi_2_Se_3_ sample was analyzed by an EDS spectrum (Figure 2e) that shows that the as-prepared sample consists of Bi and Se only, hence confirming the chemical purity of the sample. The peak corresponding to Cu in the EDS spectrum arises from the TEM grid used for preparing the TEM specimen. From the TEM analysis, it can be concluded that the well-defined and clear lattice fringes in the HRTEM image as well as the distinct rings in the SAED pattern reveal the high crystalline quality of the as-synthesized Bi_2_Se_3_ nanoflakes.

**Figure 3:**
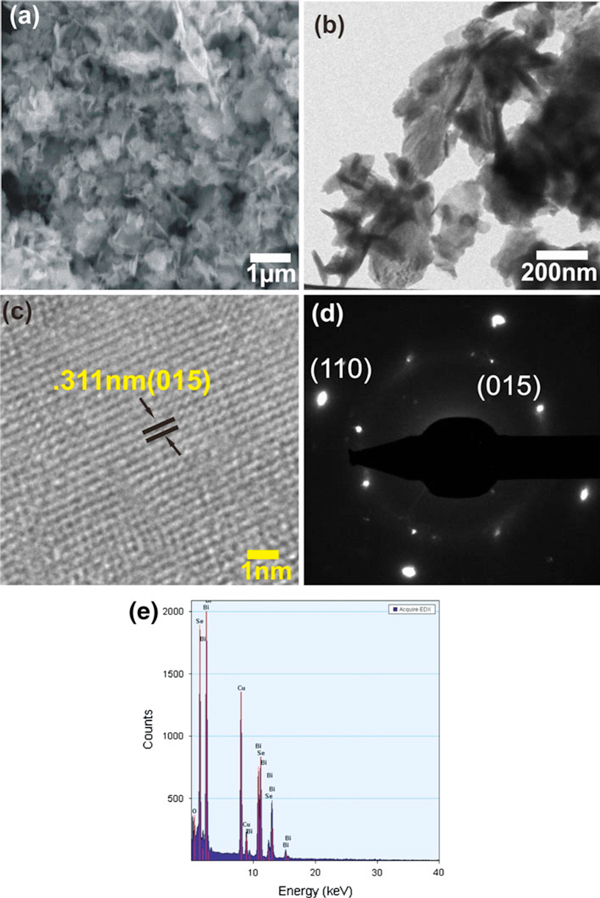
**SEM and TEM images of the as-prepared Bi_2_Se_3_ samples synthesized in DMF at 200°C for 24 h (BiSe-2)**. (**a**) SEM image, (**b**) TEM image, (**c**) HRTEM image, (**d**) SAED pattern, and (**e**) EDS pattern.

SEM and TEM images of the BiSe-2 sample are shown in Figure 3. A typical SEM image of BiSe-2 (Figure 3a) reveals the presence of the flake-like nanostructures similar in shape but slightly bigger in size when compared to the nanoflakes of BiSe-1 sample (see Figure 2a). The increase in size of the nanoflakes can be attributed to the increase in the synthesis temperature. Wang et al. [14] reported the increase in particle size of hydrothermally synthesized Bi_2_Se_3_ nanospheres from about 30 to 100 nm when the temperature was increased from 130 to 200°C. Figure 3b is a TEM image of the as-prepared sample, which shows the thin and translucent nanoflakes with the size ranging from 180 to 400 nm. Figure 3c is a HRTEM image of the BiSe-2 sample, and it shows clearly the equally spaced lattice fringes. The calculated fringe separation is 0.311 nm, which corresponds to the *d*-spacing of (015) plane of rhombohedral Bi_2_Se_3_. Figure 3d shows the SAED spot pattern that is indexed to corresponding lattice planes of rhombohedral Bi_2_Se_3_. The EDS spectrum of the sample, shown in Figure 3e, shows that the as-prepared sample consists of Bi and Se only, hence confirming the chemical purity of the sample. Clearly distinguishable lattice fringes in HRTEM image indicate the high crystallinity of the sample, and the spotty SAED pattern reveals the single-crystalline nature of the BiSe-2 sample.

### Thermoelectric Property of the Bi_2_Se_3_ Nanoparticles

**Figure 4:**
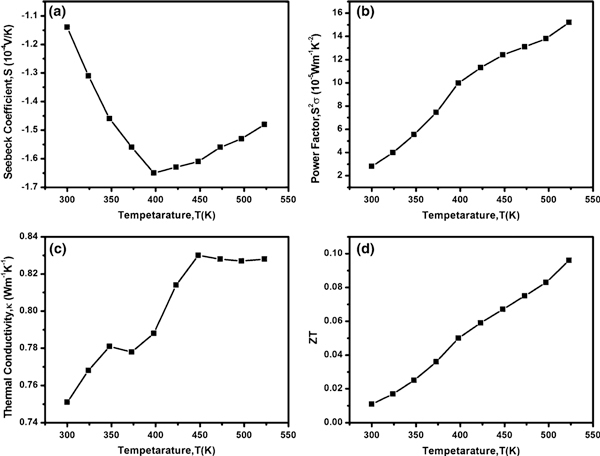
**Temperature dependence of thermoelectric properties of sample prepared in DMF at 200°C for 24 h (BiSe-2)**. (**a**) Seebeck coefficient (S) versus temperature (T), (**b**) power factor (S^2^σ) versus temperature (T), (**c**) thermal conductivity (k) versus temperature (T), and (**d**) figure of merit (ZT) versus temperature (T).

The temperature dependence of the transport properties of the BiSe-2 sample is shown in Figure 4. Figure 4a shows the plot of the Seebeck coefficient (S) versus the temperature measured in the range of 300–523 K. The negative value of the Seebeck coefficient indicates that the as-prepared Bi_2_Se_3_ nanostructures are n-type in nature. The graph shows that Seebeck coefficient increases with temperature, attains a maximum value around 400 K, and decreases. Zou et al. [26] have also reported a similar result for the Seebeck coefficient for *n*-type Bi_2_Te_3_ thin film that shows the maximum Seebeck coefficient value at about 530 K. Further study is required to understand the relationship between the Seebeck coefficient and the temperature. The result shows that the magnitude of S for BiSe-2 sample at room temperature (300 K) is about 1.15 × 10^-4^ V/K, which is about two times as much as that for the bulk Bi_2_Se_3_, 0.59 × 10^-4^ V/K, at room temperature [27]. The increase in the Seebeck coefficient arises due to the quantum confinement of electrons induced by nanostructures and is necessary for the enhancement of the thermoelectric efficiency. From the definition of ZT, the greater the magnitude of S, the larger the ZT of the material. Recent work by Lin et al. [23] reports the room temperature Seebeck coefficient of 0.84 × 10^-4^ V/K of Bi_2_Se_3_ nanoplates obtained from decomposition of single-source precursor. Figure 4b represents the temperature dependence of the power factor of BiSe-2 sample that shows that the power factor (S^2^σ) increases with the temperature. The increase in power factor with the temperature can be attributed to the increase in the electrical conductivity with temperature due to the semiconducting nature of the Bi_2_Se_3_ nanostructures. The maximum value of the power factor is 15.2 × 10^-5^ Wm^-1^K^-2^ at 523 K, and the room temperature value is 2.8 × 10^-5^ Wm^-1^K^-2^, which is comparable to the room temperature power factor value of about 7 × 10^-5^ Wm^-1^K^-2^ of Bi_2_Se_3_ nanoplates [23].The variation of thermal conductivity (κ) with temperature is shown in Figure 4c. The lowest value of the thermal conductivity, 0.751 Wm^-1^K^-1^, is recorded at room temperature, which is lower than that for the bulk Bi_2_Se_3_, 4 Wm^-1^K^-1^. This shows the significant reduction in thermal conductivity of Bi_2_Se_3_ nanostructures, which results in the enhancement of the ZT value. The reduction in thermal conductivity of Bi_2_Se_3_ nanostructures is expected due to the interface or boundary scattering of phonons in nanostructures. Figure 4d shows the plot of ZT of BiSe-2 versus temperature in the range of 300–523 K, indicating the nearly linear increase in ZT with the temperature in the given range. The maximum ZT value is 0.096 at 523 K, and the room temperature ZT value is 0.011. The thermoelectric measurement of the BiSe-2 sample reveals the promising thermoelectric property of the as-prepared Bi_2_Se_3_ nanostructures at room temperature, but the optimization of the synthesis condition is needed to further enhance its thermoelectric efficiency.

## Conclusion

Bi_2_Se_3_ nanoflakes were synthesized via solvothermal route at different synthesis conditions using DMF as solvent. The surface morphology and crystal structure of the nanoflakes were analyzed, and the results show that the as-prepared samples are rhombohedral phase of Bi_2_Se_3_. The size of the Bi_2_Se_3_ nanoflakes increases with the synthesis temperature. From the thermoelectric property measurement, the maximum ZT value of 0.096 was obtained at 523 K, and a ZT value of 0.011 was obtained at room temperature. The as-prepared Bi_2_Se_3_ nanoflakes exhibit a higher Seebeck coefficient and a low thermal conductivity compared with the bulk counterpart at room temperature, which can be attributed to their nanoscale size. The improvement on the thermoelectric property indicates the promising aspect of the as-prepared Bi_2_Se_3_ nanoflakes as a good thermoelectric material at room temperature.
